# Generation of enzymatically competent SARS‐CoV‐2 decoy receptor ACE2‐Fc in glycoengineered *Nicotiana benthamiana*

**DOI:** 10.1002/biot.202000566

**Published:** 2021-02-12

**Authors:** Alexandra Castilho, Jennifer Schwestka, Nikolaus F. Kienzl, Ulrike Vavra, Clemens Grünwald‐Gruber, Shiva Izadi, Chaitra Hiremath, Janine Niederhöfer, Elisabeth Laurent, Vanessa Monteil, Ali Mirazimi, Gerald Wirnsberger, Johannes Stadlmann, Eva Stöger, Lukas Mach, Richard Strasser

**Affiliations:** ^1^ Department of Applied Genetics and Cell Biology Institute of Plant Biotechnology and Cell Biology University of Natural Resources and Life Sciences Vienna Vienna Austria; ^2^ Department of Chemistry Institute of Biochemistry University of Natural Resources and Life Sciences Vienna Vienna Austria; ^3^ Department of Biotechnology Faculty of Agriculture Tarbiat Modares University Tehran Iran; ^4^ Apeiron Biologics AG, Campus Vienna Biocenter Vienna Austria; ^5^ Department of Biotechnology and Core Facility Biomolecular & Cellular Analysis University of Natural Resources and Life Sciences Vienna Vienna Austria; ^6^ Department of Laboratory Medicine Unit of Clinical Microbiology Karolinska Institute and Karolinska University Hospital Stockholm Sweden

**Keywords:** COVID‐19, glycosylation, posttranslational modification, recombinant protein expression, SARS‐CoV‐2

## Abstract

Human angiotensin‐converting enzyme 2 (ACE2) is the primary host cell receptor for severe acute respiratory syndrome coronavirus 2 (SARS‐CoV‐2) binding and cell entry. Administration of high concentrations of soluble ACE2 can be utilized as a decoy to block the interaction of the virus with cellular ACE2 receptors and potentially be used as a strategy for treatment or prevention of coronavirus disease 2019. Human ACE2 is heavily glycosylated and its glycans impact on binding to the SARS‐CoV‐2 spike protein and virus infectivity. Here, we describe the production of a recombinant soluble ACE2‐fragment crystallizable (Fc) variant in glycoengineered *Nicotiana benthamiana*. Our data reveal that the produced dimeric ACE2‐Fc variant is glycosylated with mainly complex human‐type *N*‐glycans and functional with regard to enzyme activity, affinity to the SARS‐CoV‐2 receptor‐binding domain, and wild‐type virus neutralization.

## Graphical abstract

Human angiotensin‐converting enzyme 2 (ACE2) is the primary receptor for SARS‐CoV‐2 cell entry and recombinant ACE2 variants have great therapeutic potential to treat or prevent COVID‐19. Here, we show that a glycosylated recombinant ACE2‐Fc fusion protein can be produced in glycoengineered *Nicotiana benthamiana* with favorable glycosylation.

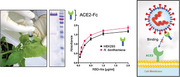

**Abbreviations**
ACE2: angiotensin‐converting enzyme 2
COVID‐19: coronavirus disease 2019
Fc: fragment crystallizable
MALS: multi‐angle light scattering
RBD: receptor‐binding domain
SARS‐CoV‐2: severe acute respiratory syndrome coronavirus 2
SEC: size‐exclusion chromatography

## 1 INTRODUCTION

Zoonotic viruses are a constant emerging threat and potent strategies to stop the spread of infectious pathogens such as severe acute respiratory syndrome coronavirus 2 (SARS‐CoV‐2), the causative pathogen of coronavirus disease 2019 (COVID‐19), and disease progression are of utmost importance. Various therapeutic avenues are currently developed and in clinical studies to treat and prevent COVID‐19. Angiotensin‐converting enzyme 2 (ACE2), a metallopeptidase of the renin‐angiotensin system involved in maintaining blood pressure homeostasis, is the primary host cell receptor for SARS‐CoV‐2 binding and cell entry.^[^ ^1^ ^]^ Administration of high concentrations of recombinant soluble human ACE2 reduces virus entry into target host cells by competition with cellular ACE2 for binding to the SARS‐CoV‐2 spike protein.^[^ ^2^ ^]^ A drawback of soluble ACE2 is the short plasma half‐life that can, however, be overcome by fusion to the fragment crystallizable (Fc) of human immunoglobulin G.^[^ ^3^ ^]^ A recombinant ACE2‐Fc fusion protein efficiently neutralized pseudovirions pseudotyped with SARS‐CoV‐2 spike protein.^[^ ^4^ ^]^ Since ACE2 is also the cell entry receptor for SARS‐CoV‐1, recombinant ACE2‐Fc and engineered high‐affinity variants thereof do not only have therapeutic potential to prevent SARS‐CoV‐2 infections but are also candidates for preventive treatments of future coronavirus outbreaks.^[^ ^5^ ^]^ For the ongoing pandemic as well as for emerging viruses, it is highly important to establish flexible production systems for recombinant protein therapeutics such as ACE2‐Fc that are required worldwide in high demands. In addition to treatment and prophylaxis, different ACE2 variants are essential components of diagnostic assays and test kits, for example, to characterize neutralizing antibodies or to screen for novel inhibitors that interfere with the binding of SARS‐CoV‐2 to ACE2. Since several years, plants are increasingly used as platforms to produce diagnostic reagents and pharmaceutical proteins. In this report, we demonstrate the production of a functional recombinant ACE2‐Fc in glycoengineered plants.

## 2 MATERIAL AND METHODS

### 2.1 Cloning

A DNA fragment coding for amino acids 18–615 of human ACE2 and codon optimized for expression in *Nicotiana benthamiana* (Thermo Fisher Scientific) was cloned into the *Bsa*I sites of the magnICON vector pICHα26211 carrying the coding sequence for the barley α‐amylase signal peptide and the Fc‐domain of human IgG1.^[^ ^6^ ^]^

### 2.2 ACE2‐Fc expression, purification, and characterization

ACE2‐Fc was produced by agrobacterium‐mediated transient expression in leaves of *N. benthamiana* ΔXT/FT. Four days after infiltration, a total soluble protein extract was prepared and clarified as described previously.^[^ ^7^, ^8^ ^]^ ACE2‐Fc was purified by affinity chromatography using a 5 mL HiTrap Protein A column (Cytiva) and 0.1 M glycine‐HCl (pH 3.5) for elution. Eluate fractions were immediately neutralized using 2 M Tris (pH 12.0), pooled, concentrated (Amicon Ultra‐0.5 Centrifugal Filter Units, Millipore) and dialyzed against PBS (pH 7.4) at 4°C overnight using SnakeSkin Dialysis Tubing (Thermo Fisher Scientific). Affinity‐purified ACE2‐Fc was subjected to size‐exclusion chromatography (SEC) using a HiLoad 16/600 Superdex 200 pg column (Cytiva) equilibrated with PBS (pH 7.4) for polishing. SEC‐multi‐angle light scattering (SEC‐MALS) analysis was carried out as described recently.^[^ ^9^ ^]^ Production of ACE2‐Fc in HEK293 cells was performed as described previously.^[^ ^5^ ^]^ ACE2‐Fc separated by SDS‐PAGE was detected by Coomassie Blue staining or immunoblotting with anti‐human IgG (H+L)‐horseradish peroxidase antibody (Promega). For deglycosylation, samples were incubated with Endoglycosidase H (Endo H) or peptide‐*N*‐glycosidase F (PNGase F) (New England Biolabs) according to the manufacturer's instructions.

### 2.3 Binding assays

ACE2‐receptor‐binding domain (RBD) interaction studies were performed on an Octet RED96e system using high precision streptavidin biosensors (ForteBio). ACE2‐Fc was biotinylated using the EZ‐Link Sulfo‐NHS‐LC Biotin kit (Thermo Fisher Scientific) and purified using PD‐10 desalting columns (Cytiva). All assays were conducted in PBS supplemented with 0.05% v/v Tween 20 and 0.1% w/v BSA (PBST‐BSA) at 25°C with the plate shaking at 1000 rpm. The biosensors were first equilibrated in PBST‐BSA and then dipped into a 34 nM solution of the respective biotinylated capture molecule. To determine K_d_ values, titration of HEK293‐produced RBD was performed to cover a broad concentration range around the respective K_d_ value.^[^ ^10^ ^]^ To record association rates, ACE2‐Fc‐loaded biosensors were submerged either into two‐fold (200–6.25 nM) or three‐fold (200–0.8 nM) serial dilutions of RBD for 600 s. For dissociation, the biosensors were dipped into PBST‐BSA for 100 s. Each experiment was performed in triplicates. Data were evaluated using the Octet data analysis software version 11.1.1.39. ELISA assays were carried out as described.^[^ ^10^ ^]^

### 2.4 ACE2 activity assays

Hydrolysis of (7‐methoxycoumarin‐4‐yl)acetyl‐Ala‐Pro‐Lys(2,4‐dinitrophenyl) (Mca‐APK‐DNP; Bachem) was used to quantify ACE2 peptidase activity. An ACE2‐Fc dilution series (0.2–1 nM) was incubated in 50 mM MES, 300 mM NaCl, 10 μM ZnCl_2_, 0.01% Brij‐35 (pH 6.5) containing 100 μM Mca‐APK‐DNP at 37°C and the increase in fluorescence (320 nm excitation/430 nm emission) was monitored over time.

### 2.5 Wild‐type virus neutralization assays

SARS‐CoV‐2 neutralization by ACE2‐Fc was assayed by qRT‐PCR analysis of virus‐exposed Vero E6 cells as described.^[^ ^2^ ^]^ The wild‐type SARS‐CoV‐2 strain used was isolated from an infected Swedish individual (GenBank accession number MT093571).

### 2.6 Glycopeptide analysis

SEC‐purified ACE2‐Fc was sequentially digested with chymotrypsin and trypsin and the glycopeptides were subjected to LC‐ESI‐MS analysis as described.^[^ ^8^ ^]^

## 3 RESULTS

### 3.1 Transient expression of human ACE2‐Fc in plants

**FIGURE 1:**
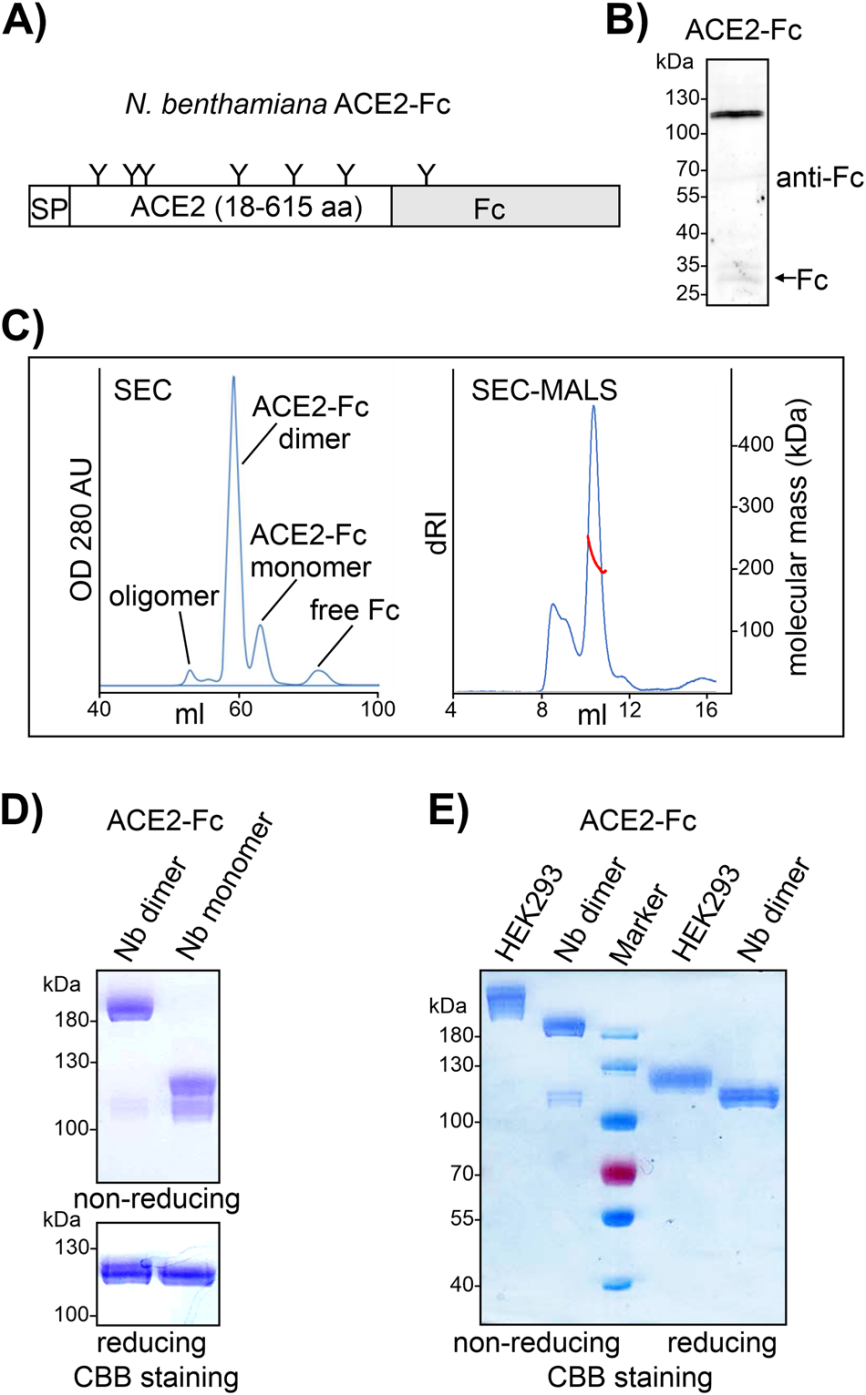
Expression of ACE2‐Fc in *N. benthamiana*. (A) Illustration of the expressed human ACE2‐Fc protein. *N*‐glycans (“Y”) are indicated. SP, signal peptide from barley α‐amylase. (B) Immunoblot detection of ACE2‐Fc in total protein extracts from ΔXT/FT *N. benthamiana* extracted 4 days after infiltration. (C) Left panel: SEC purification of affinity‐purified ACE2‐Fc. Right panel: determination of the native molecular mass of dimeric ACE2‐Fc by SEC‐MALS. Blue trace: protein concentration monitored by differential refractive index (dRI). Red trace: MALS estimates of native molecular mass. (D) SDS‐PAGE of *N. benthamiana*‐produced dimeric and monomeric ACE2‐Fc. CBB, Coomassie Brilliant Blue staining. (E) SDS‐PAGE of HEK293‐derived and plant‐produced ACE2‐Fc

Recombinant ACE2‐Fc was produced in leaves from glycoengineered ΔXT/FT *N. benthamiana* using a viral expression vector (Figure 1A).^[^ ^7^ ^]^ In total soluble protein extracts, the main band of ≈110 kDa corresponds to full‐length glycosylated ACE2‐Fc (Figure 1B). A faint band of ≈35 kDa was also detected, which indicates limited proteolytic release of the Fc domain. ACE2‐Fc was purified from total soluble protein extracts by protein A affinity chromatography and then subjected to preparative SEC. In addition to the slowly eluting peak containing cleaved Fc (about 5% of the affinity‐purified material), we detected three peaks that likely correspond to intact monomeric (10%), dimeric (80%), and oligomeric (5%) ACE2‐Fc (Figure 1C). The dimer fraction was isolated and subjected to SEC analysis combined with MALS detection to determine its native molecular mass. The experimentally obtained estimate of 213 kDa is in very good agreement with the native molecular mass expected for a fully glycosylated ACE2‐Fc dimer (209 kDa), assuming a molecular weight of 1.5 kDa for each of its seven *N*‐glycans per subunit (Figure 1A). SDS‐PAGE under non‐reducing conditions confirmed the successful separation of monomeric and dimeric ACE2‐Fc by preparative SEC (Figure 1D). Under reducing conditions, purified dimeric and monomeric ACE2‐Fc variants migrated at the same position between 100 and 130 kDa. Plant‐derived and HEK293‐produced ACE2‐Fc differ in the linker and Fc‐hinge region but have a similar calculated molecular weight (93.9 vs. 94.9 kDa) and the same number of *N*‐glycosylation sites per polypeptide chain (Table S1, Supporting Information). The reduced mobility of HEK293 ACE2‐Fc (Figure 1E) is likely derived from differences in *N*‐glycan processing.^[^ ^11^ ^]^ After protein A purification the ACE2‐Fc yield was ≈80 μg g^−1^ leaf fresh weight when extracted 4 days after infiltration, with 50 μg g^−1^ recovered as dimer after SEC purification.

### 3.2 The ACE2 peptidase domain of plant‐produced recombinant ACE2‐Fc is functional

**FIGURE 2:**
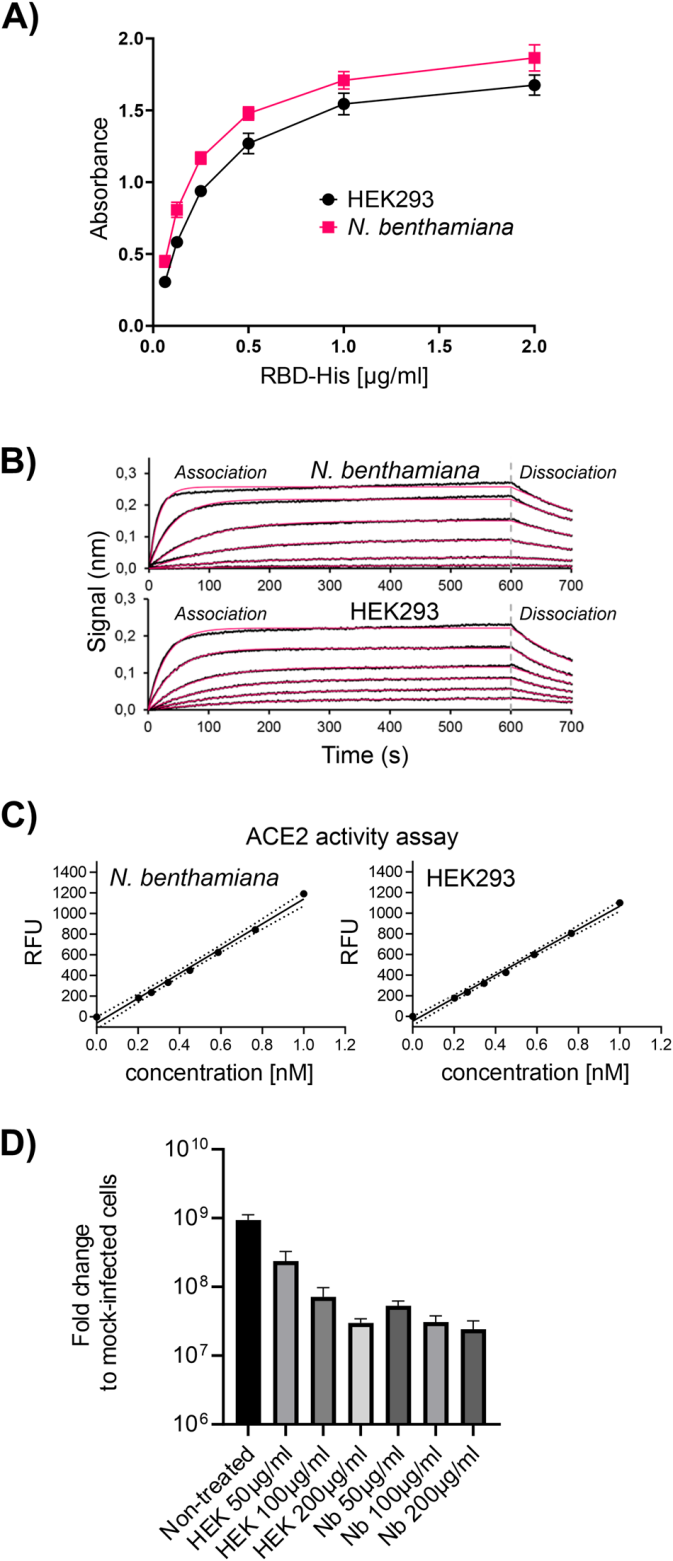
The ACE2 domain of plant‐produced ACE2‐Fc is functional. (A) ELISA to detect the binding of ACE2‐Fc to RBD‐His produced in HEK293 cells. Data are presented as mean ± SD (*n* = 3). (B) Binding of RBD‐His to immobilized *N. benthamiana* ACE2‐Fc monitored by biolayer interferometry. (C) ACE2 activity assays. (D) SARS‐CoV‐2 neutralization assay. Virus was mixed with different concentrations of recombinant ACE2‐Fc and added to Vero E6 cells. Viral RNA was extracted after 15 h and quantified by qRT‐PCR. Data are presented as mean ± SD (*n* = 3)

To examine whether the peptidase domain of ACE2‐Fc is correctly folded and capable of interaction with the RBD of SARS‐CoV‐2, we first characterized the binding of HEK293‐derived RBD to immobilized ACE2‐Fc using a sandwich ELISA. Plates were coated with anti‐human Fc to capture ACE2‐Fc and incubated with different concentrations of hexahistidine‐tagged RBD. In the ELISA, plant‐derived ACE2‐Fc displayed a slightly better binding than HEK293‐produced ACE2‐Fc (Figure 2A). In line with this finding, the binding affinity to RBD determined by biolayer interferometry was increased for plant‐derived ACE2‐Fc (FIGURE 2B) with a K_d_ of 10.1 ± 0.2 nM compared to a K_d_ of 22.8 ± 4.1 nM for ACE2‐Fc produced in HEK293 cells.

ACE2 is not only the primary receptor for SARS‐CoV‐2, but also a zinc‐metallopeptidase that converts the octapeptide angiotensin II to angiotensin 1–7. The peptidase domain is incorporated in our expressed ACE2‐Fc variant and thus we tested ACE2‐Fc for proteolytic activity using a fluorogenic peptide substrate. The plant‐derived ACE2‐Fc efficiently hydrolyzed the synthetic peptide with equal efficiency as HEK‐produced ACE2‐Fc (Figure 2C). In a wild‐type SARS‐CoV‐2 virus neutralization assay, *N. benthamiana* produced ACE2‐Fc was at least equally effective as HEK293 ACE2‐Fc (Figure 2D). Together these data show that the recombinant ACE2 peptidase domain from ACE2‐Fc is correctly folded and fully functional when produced in plants.

### 3.3 ACE2‐Fc is glycosylated with complex *N*‐glycans

**FIGURE 3:**
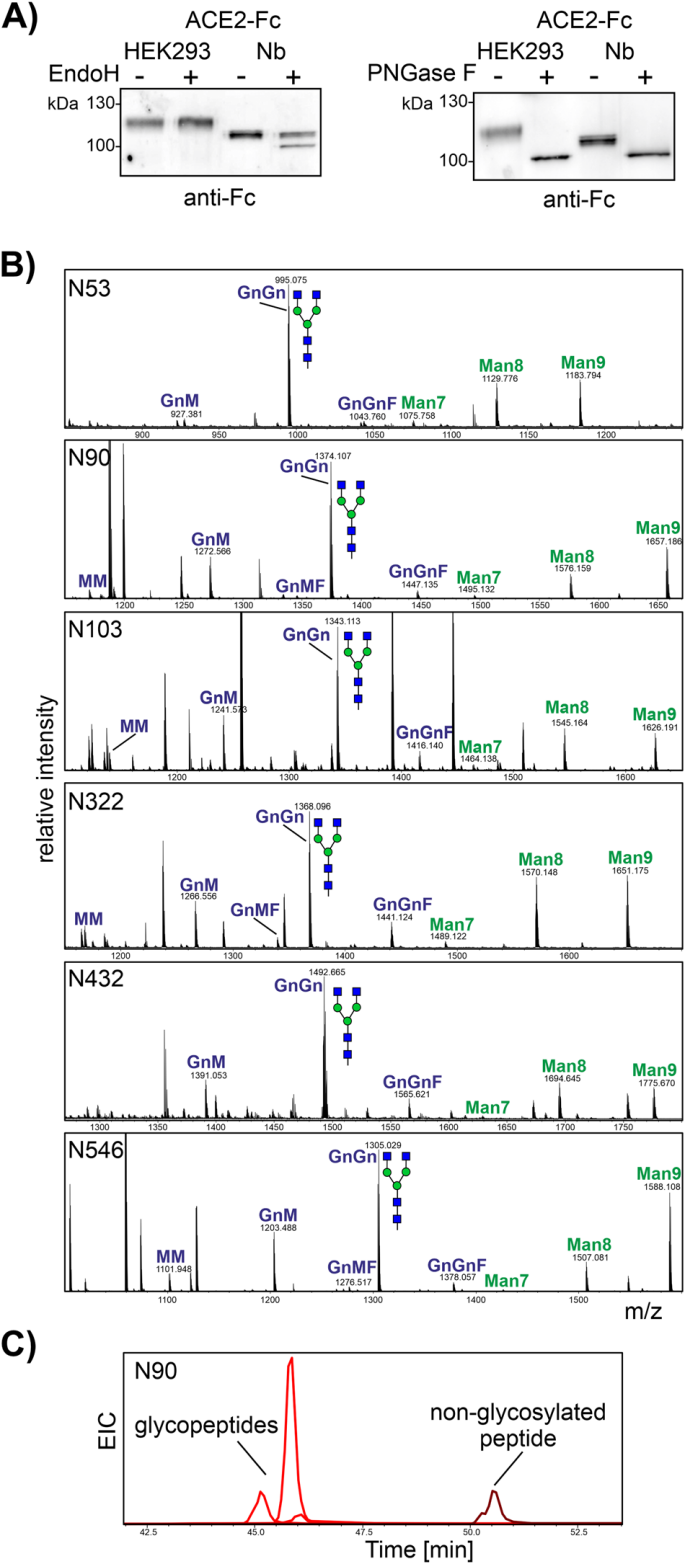
ACE2‐Fc glycosylation. (A) Immunoblot analysis of Endo H and PNGase F digested ACE2‐Fc purified from *N. benthamiana* and HEK293 cells. (B) MS spectra [M+2H]^2+^ of glycopeptides from plant‐produced ACE2‐Fc. The assigned *N*‐glycan structures were labeled according to the ProGlycAn nomenclature (http://www.proglycan.com/). A cartoon illustration highlights the main glycan structure detected for each peptide (http://www.functionalglycomics.org/). (C) Extracted ion chromatogram (EIC) of the most abundant glycopeptides and the non‐glycosylated peptide found on glycosylation site N90 of ACE2‐Fc expressed in *N. benthamiana*

Human ACE2 is heavily glycosylated and *N*‐glycans play a role for the interaction with the RBD of SARS‐CoV‐2 spike protein.^[^ ^11^ ^]^ The expressed ACE2 peptidase domain harbors six *N*‐glycosylation sites (Figure 1A) and all of them are glycosylated in mammalian cells.^[^ ^11^ ^]^ The reduced mobility on SDS‐PAGE and the determined native molecular mass suggests that the plant‐produced variant is also heavily glycosylated. Moreover, SDS‐PAGE of plant‐produced ACE2‐Fc under reducing conditions revealed three closely migrating bands indicative of non‐uniform *N*‐glycosylation or *N*‐glycan processing. When we treated purified ACE2‐Fc with PNGase F, a clear shift in mobility was detected and the deglycosylated forms from HEK293 cells and *N. benthamiana* co‐migrated (Figure 3A). Upon Endo H digestion, no shift was visible for HEK293‐produced ACE2‐Fc, but a portion of the plant produced ACE2‐Fc displayed faster migration indicating the presence of small amounts of Endo H sensitive oligomannosidic *N*‐glycans.

To characterize ACE2‐Fc *N*‐glycosylation in a site‐specific manner, we digested purified dimeric ACE2‐Fc and subjected the glycopeptides to LC‐ESI‐MS analysis. *N*‐glycans could be detected on all sites, with the *N*‐glycan profiles of all six glycopeptides being remarkably similar. The major peak corresponded to the complex *N*‐glycan GlcNAc_2_Man_3_GlcNAc_2_ (GnGn) (Figure 3B). In addition, smaller peaks due to oligomannosidic *N*‐glycans (Man7—Man_7_GlcNAc_2_, Man8—Man_8_GlcNAc_2_, and Man9—Man_9_GlcNAc_2_) were also present. While five sites were completely glycosylated, N90 displayed only partial occupancy (92 ± 3.5%, mean ± SD, *n* = 3) (Figure 3C). By contrast, HEK293‐derived ACE2‐Fc analyzed by the same glycoproteomic approach is fully glycosylated on all six *N*‐glycosylation sites. As described by a previous report for soluble ACE2, the *N*‐glycans from HEK293‐produced ACE2‐Fc are more extensively processed and display increased microheterogeneity compared to plant‐produced ACE2‐Fc (Figure S1, Supporting Information).^[^ ^11^ ^]^ Engineered ACE2 lacking the *N*‐glycan at N90 has a two‐fold higher affinity to RBD.^[^ ^5^ ^]^ It is therefore plausible that the higher affinity of ACE2‐Fc from *N. benthamiana* for RBD results to some extent from the partial underglycosylation at this position. Alternatively, the less processed complex *N*‐glycans lacking galactose and sialic acid may be favorable for RBD binding. These possibilities will be addressed in future studies.

## 4 DISCUSSION

Transient plant expression systems are highly suitable for fast and scalable manufacturing of recombinant proteins and several plant biotechnology companies utilize this approach with *N. benthamiana* as a host to produce COVID‐19 vaccine candidates that have entered clinical trials. Here, we show that functional recombinant ACE2‐Fc with high binding affinity for SARS‐CoV‐2 RBD can be efficiently produced in plants and may be used as receptor decoy to treat coronavirus infections in humans.

Besides supporting fast and efficient purification from soluble protein extracts, the fusion of the Fc domain to the ACE2 peptidase domain is supposed to extend the in vivo half‐life in therapeutic applications through binding to the neonatal Fc receptor.^[^ ^12^ ^]^ The Fc domain enables also interactions with other Fc receptors present on immune cells. These interactions trigger Fc‐mediated effector functions including mechanisms for clearance of viruses and virus‐infected cells. Fusion to the full‐length Fc region of human IgG1 retains cysteine residues required for homodimer formation. From a previous study with EPO‐Fc, we know, however, that considerable amounts of the Fc domain are cleaved *in planta* resulting in a mixture of full‐length fusion protein and the cleaved Fc domain.^[^ ^13^ ^]^ Moreover, we have observed that the hinge region is particularly susceptible to cleavage by endogenous plant proteases.^[^ ^14^ ^]^ To minimize proteolytic degradation of ACE2‐Fc, we have therefore replaced the processing‐prone part of the hinge region with a protease‐resistant linker. Although the CPPC sequence from the hinge region involved in dimerization is missing in our fusion protein, the vast majority of ACE2‐Fc was still present as a dimer and bound to SARS‐CoV‐2 RBD at least as well as ACE2‐Fc produced in HEK293 cells. Human recombinant soluble ACE2 has been used successfully to treat a severe COVID‐19 patient.^[^ ^15^ ^]^ Recombinant ACE2‐Fc could be used in a similar manner. While certain Fc‐receptor mediated effector functions could further enhance its anti‐viral activities, there are concerns that the Fc portion of ACE2‐Fc may also pose a risk due to Fcγ receptor‐mediated functions like antibody‐dependent enhancement.^[^ ^16^ ^]^ Whether the presence of the human Fc domain in ACE2‐Fc is beneficial or has adverse consequences in vivo cannot be predicted and has to be explored in further studies using FcγR humanized mice and testing in humans.^[^ ^17^ ^]^

## CONFLICT OF INTEREST

The authors declare no conflict of interest. Gerald Wirnsberger and Janine Niederhöfer are employees of Apeiron Biologics AG.

## AUTHOR CONTRIBUTIONS

**Alexandra Castilho**: Conceptualization; funding acquisition; investigation; methodology; supervision; writing – original draft; writing – review and editing. **Jennifer Schwestka**: Investigation; writing – review and editing. **Nikolaus F. Kienzl**: Investigation; writing – review and editing. **Ulrike Vavra**: Investigation; writing – review and editing. **Clemens Grünwald‐Gruber**: Investigation; writing – review and editing. **Shiva Izadi**: Investigation; writing – review and editing. **Chaitra Hiremath**: Investigation; writing – review and editing. **Janine Niederhöfer**: Investigation; writing – review and editing. **Elisabeth Laurent**: Investigation; methodology; writing – review and editing. **Vanessa Monteil**: Investigation; methodology; writing – review and editing. **Ali Mirazimi**: Methodology; resources; supervision; writing – review and editing. **Gerald Wirnsberger**: Methodology; resources; supervision; writing – review and editing. **Johannes Stadlmann**: Methodology; supervision; writing – review and editing. **Eva Stöger**: Methodology; supervision; writing – review and editing. **Lukas Mach**: Conceptualization; funding acquisition; methodology; supervision; writing – original draft; writing – review and editing. **Richard Strasser**: Conceptualization; funding acquisition; methodology; project administration; resources; supervision; writing – original draft; writing – review and editing.

## Supporting information



Supplementary information

## Data Availability

The data that support the findings of this study are available from the corresponding author upon reasonable request.

## References

[1] Hoffmann, M. , Kleine‐Weber, H. , Schroeder, S. , Krüger, N. , Herrler, T. , Erichsen, S. , … Pöhlmann, S. (2020). SARS‐CoV‐2 cell entry depends on ACE2 and TMPRSS2 and is blocked by a clinically proven protease inhibitor. Cell, 181, 271‐280.e8. 32142651 10.1016/j.cell.2020.02.052 PMC7102627

[2] Monteil, V. , Kwon, H. , Prado, P. , Hagelkrüys, A. , Wimmer, R. A. , Stahl, M. , … Penninger, J. M. (2020). Inhibition of SARS‐CoV‐2 infections in engineered human tissues using clinical‐grade soluble human ACE2. Cell, 181, 905‐913.e7. 32333836 10.1016/j.cell.2020.04.004 PMC7181998

[3] Haschke, M. , Schuster, M. , Poglitsch, M. , Loibner, H. , Salzberg, M. , Bruggisser, M. , … Krähenbühl, S. (2013). Pharmacokinetics and pharmacodynamics of recombinant human angiotensin‐converting enzyme 2 in healthy human subjects. Clinical Pharmacokinetics, 52, 783–792. 23681967 10.1007/s40262-013-0072-7

[4] Lei, C. , Qian, K. , Li, T. , Zhang, S. , Fu, W. , Ding, M. , & Hu, S. (2020). Neutralization of SARS‐CoV‐2 spike pseudotyped virus by recombinant ACE2‐Ig. Nature Communications, 11, 2070. 10.1038/s41467-020-16048-4 PMC7265355 32332765

[5] Chan, K. K. , Dorosky, D. , Sharma, P. , Abbasi, S. A. , Dye, J. M. , Kranz, D. M. , … Procko, E. (2020). Engineering human ACE2 to optimize binding to the spike protein of SARS coronavirus 2. Science, 369, 1261–1265. 32753553 10.1126/science.abc0870 PMC7574912

[6] Schneider, J. D. , Castilho, A. , Neumann, L. , Altmann, F. , Loos, A. , Kannan, L. , … Steinkellner, H. (2014). Expression of human butyrylcholinesterase with an engineered glycosylation profile resembling the plasma‐derived orthologue. Biotechnology Journal, 9, 501–510. 24130173 10.1002/biot.201300229 PMC3975692

[7] Strasser, R. , Stadlmann, J. , Schähs, M. , Stiegler, G. , Quendler, H. , Mach, L. , … Steinkellner, H. (2008). Generation of glyco‐engineered *Nicotiana benthamiana* for the production of monoclonal antibodies with a homogeneous human‐like N‐glycan structure. Plant Biotechnology Journal, 6, 392–402. 18346095 10.1111/j.1467-7652.2008.00330.x

[8] Göritzer, K. , Maresch, D. , Altmann, F. , Obinger, C. , & Strasser, R. (2017). Exploring site‐specific N‐glycosylation of HEK293 and plant‐produced human IgA isotypes. Journal of Proteome Research, 16, 2560–2570. 28516782 10.1021/acs.jproteome.7b00121 PMC5504489

[9] Puchol Tarazona, A. A. , Lobner, E. , Taubenschmid, Y. , Paireder, M. , Torres Acosta, J. A. , Göritzer, K. , … Mach, L. (2020). Steric accessibility of the cleavage sites dictates the proteolytic vulnerability of the anti‐HIV‐1 antibodies 2F5, 2G12, and PG9 in plants. Biotechnology Journal, 15, 1900308. 10.1002/biot.201900308 31657528

[10] Stadlbauer, D. , Amanat, F. , Chromikova, V. , Jiang, K. , Strohmeier, S. , Arunkumar, G. A. , … Krammer, F. (2020). SARS‐CoV‐2 seroconversion in humans: A detailed protocol for a serological assay, antigen production, and test setup. Current Protocols in Microbiology, 57, e100. 32302069 10.1002/cpmc.100 PMC7235504

[11] Zhao, P. , Praissman, J. L. , Grant, O. C. , Cai, Y. , Xiao, T. , Rosenbalm, K. E. , … Wells, L. (2020). Virus‐receptor interactions of glycosylated SARS‐CoV‐2 spike and human ACE2 receptor. Cell Host & Microbe, 28, 586–601. 32841605 10.1016/j.chom.2020.08.004 PMC7443692

[12] Liu, P. , Wysocki, J. , Souma, T. , Ye, M. , Ramirez, V. , Zhou, B. , … Jin, J. (2018). Novel ACE2‐Fc chimeric fusion provides long‐lasting hypertension control and organ protection in mouse models of systemic renin angiotensin system activation. Kidney International, 94, 114–125.2969106410.1016/j.kint.2018.01.029

[13] Castilho, A. , Neumann, L. , Gattinger, P. , Strasser, R. , Vorauer‐Uhl, K. , Sterovsky, T. , … Steinkellner, H. (2013). Generation of biologically active multi‐sialylated recombinant human EPOFc in plants. PLoS One, 8, e54836. 23372778 10.1371/journal.pone.0054836 PMC3555983

[14] Niemer, M. , Mehofer, U. , Torres Acosta, J. A. , Verdianz, M. , Henkel, T. , Loos, A. , … Mach, L. (2014). The human anti‐HIV antibodies 2F5, 2G12, and PG9 differ in their susceptibility to proteolytic degradation: Down‐regulation of endogenous serine and cysteine proteinase activities could improve antibody production in plant‐based expression platforms. Biotechnology Journal, 9, 493–500. 24478053 10.1002/biot.201300207 PMC4162989

[15] Zoufaly, A. , Poglitsch, M. , Aberle, J. H. , Hoepler, W. , Seitz, T. , Traugott, M. , … Penninger, J. M. (2020). Human recombinant soluble ACE2 in severe COVID‐19. The Lancet. Respiratory Medicine, 8, 1154–1158. 33131609 10.1016/S2213-2600(20)30418-5 PMC7515587

[16] Tada, T. , Fan, C. , Chen, J. S. , Kaur, R. , Stapleford, K. A. , Gristick, H. , … Landau, N. R. (2020). An ACE2 microbody containing a single immunoglobulin Fc domain is a potent inhibitor of SARS‐CoV‐2. Cell Reports, 33, 108528. 33326798 10.1016/j.celrep.2020.108528 PMC7705358

[17] Bournazos, S. , Gupta, A. , & Ravetch, J. V. (2020). The role of IgG Fc receptors in antibody‐dependent enhancement. Nature Reviews Immunology, 20, 633–643. 10.1038/s41577-020-00410-0 PMC7418887 32782358

