# Supplementary material for: Generation of enzymatically competent SARS‐CoV‐2 decoy receptor ACE2‐Fc in glycoengineered Nicotiana benthamiana
Source: Biotechnol J. 2021 Feb 12;16(6):2000566. doi: 10.1002/biot.202000566 (PMC7995010; doi:10.1002/biot.202000566)
Supplement: Supplementary file 1 — Supplementary information [file BIOT-16-0-s001.pdf]

Supporting Information

Generation of enzymatically competent SARS-CoV-2 decoy receptor ACE2-Fc in glycoengineered *Nicotiana benthamiana*

Alexandra Castilho[1], Jennifer Schwestka[1], Nikolaus F. Kienzl[1], Ulrike Vavra[1], Clemens Grünwald-Gruber[2], Shiva Izadi[1,5], Chaitra Hiremath[1], Janine Niederhöfer[4], Elisabeth Laurent[3], Vanessa Monteil[6], Ali Mirazimi[6], Gerald Wirnsberger[4], Johannes Stadlmann[2], Eva Stöger[1], Lukas Mach[1], Richard Strasser[1]

[1]Department of Applied Genetics and Cell Biology, Institute of Plant Biotechnology and Cell Biology, University of Natural Resources and Life Sciences Vienna, Vienna, Austria
[2]Department of Chemistry, Institute of Biochemistry, University of Natural Resources and Life Sciences Vienna, Vienna, Austria
[3]Department of Biotechnology and Core Facility Biomolecular & Cellular Analysis, University of Natural Resources and Life Sciences Vienna, Austria
[4]Apeiron Biologics AG, Campus Vienna Biocenter, Vienna, Austria
[5]Department of Biotechnology, Faculty of Agriculture, Tarbiat Modares University, Tehran, Iran
[6]Karolinska Institute and Karolinska University Hospital, Department of Laboratory Medicine, Unit of Clinical Microbiology, Stockholm, Sweden

Correspondence: Dr. Richard Strasser, Department of Applied Genetics and Cell Biology, University of Natural Resources and Life Sciences Vienna, Muthgasse 18, A-1190 Vienna, Austria
E-mail: richard.strasser@boku.ac.at

Table S1. Comparison of *N. benthamiana* ACE2-Fc and HEK293 ACE2-Fc sequences. Both fusion proteins contain the peptidase domain of human ACE2 (Q9BYF1, amino acids 18-615). The signal peptides (*N. benthamiana*: barley α-amylase; HEK293: native ACE2 sequence) are highlighted in green, the N-glycosylation sites in magenta, linker sequences in light blue and the human Fc region in yellow.

>*N. benthamiana* ACE2-Fc
MANKHMSLSLFIVLLGLSCSLASGQSTIEEQAKTFLDKFNHEAEDLFYQSSLASWNYNTNITEENVQNMNN
AGDKWSAFLKEQSTLAQMYPLQEIQNLTVKLQLQALQQNGSSVLSEDKSKRLNTILNTMSTIYSTGKVCNP
DNPQECLLLEPGLNEIMANSLDYNERLWAWESWRSEVGKQLRPLYEEYVVLKNEMARANHYEDYGDYWRGD
YEVNGVDGYDYSRGQLIEDVEHTFEEIKPLYEHLHAYVRAKLMNAYPSYISPIGCLPAHLLGDMWGRFWTN
LYSLTVPFGQKPNIDVTDAMVDQAWDAQRIFKEAEKFFVSVGLPNMTQGFWENSMLTDPGNVQKAVCHPTA
WDLGKGDFRILMCTKVTMDDFLTAHHEMGHIQYDMAYAAQPFLLRNGANEGFHEAVGEIMSLSAATPKHLK
SIGLLSPDFQEDNETEINFLLKQALTIVGTLPFTYMLEKWRWMVFKGEIPKDQWMKKWWEMKREIVGVVEP
VPHDETYCDPASLFHVSNDYSFIRYYTRTLYQFQFQEALCQAAKHEGPLHKCDISNSTEAGQKLFNMLRLG
KSEPWTLALENVVGAKNMNVRPLLNYFEPLFTWLKDQNKNSFVGWSTDWSPYADGGGGSGGGGSLGGPSVF
LFPPKPKDTLMISRTPEVTCVVVDVSHEDPEVKFNWYVDGVEVHNAKTKPREEQYNSTYRVVSVLTVLHQD
WLNGKEYKCKVSNKALPAPIEKTISKAKGQPREPQVYTLPPSRDELTKNQVSLTCLVKGFYPSDIAVEWES
NGQPENNYKTTPPVLDSDGSFFLYSKLTVDKSRWQQGNVFSCSVMHEALHNHYTQKSLSLSPGK

>HEK293 ACE2-Fc
MSSSSWLLLSLVAVTAAQSTIEEQAKTFLDKFNHEAEDLFYQSSLASWNYNTNITEENVQNMNNAGDKWSA
FLKEQSTLAQMYPLQEIQNLTVKLQLQALQQNGSSVLSEDKSKRLNTILNTMSTIYSTGKVCNPDNPQECL
LLEPGLNEIMANSLDYNERLWAWESWRSEVGKQLRPLYEEYVVLKNEMARANHYEDYGDYWRGDYEVNGVD
GYDYSRGQLIEDVEHTFEEIKPLYEHLHAYVRAKLMNAYPSYISPIGCLPAHLLGDMWGRFWTNLYSLTVP
FGQKPNIDVTDAMVDQAWDAQRIFKEAEKFFVSVGLPNMTQGFWENSMLTDPGNVQKAVCHPTAWDLGKGD
FRILMCTKVTMDDFLTAHHEMGHIQYDMAYAAQPFLLRNGANEGFHEAVGEIMSLSAATPKHLKSIGLLSP
DFQEDNETEINFLLKQALTIVGTLPFTYMLEKWRWMVFKGEIPKDQWMKKWWEMKREIVGVVEPVPHDETY
CDPASLFHVSNDYSFIRYYTRTLYQFQFQEALCQAAKHEGPLHKCDISNSTEAGQKLFNMLRLGKSEPWTL
ALENVVGAKNMNVRPLLNYFEPLFTWLKDQNKNSFVGWSTDWSPYADGSDKTHTCPPCPAPELLGGPSVFL
FPPKPKDTLMISRTPEVTCVVVDVSHEDPEVKFNWYVDGVEVHNAKTKPREEQYNSTYRVVSVLTVLHQDW
LNGKEYKCKVSNKALPAPIEKTISKAKGQPREPQVYTLPPSREEMTKNQVSLTCLVKGFYPSDIAVEWESN
GQPENNYKTTPPVLDSDGSFFLYSKLTVDKSRWQQGNVFSCSVMHEALHNHYTQKSLSLSPGK

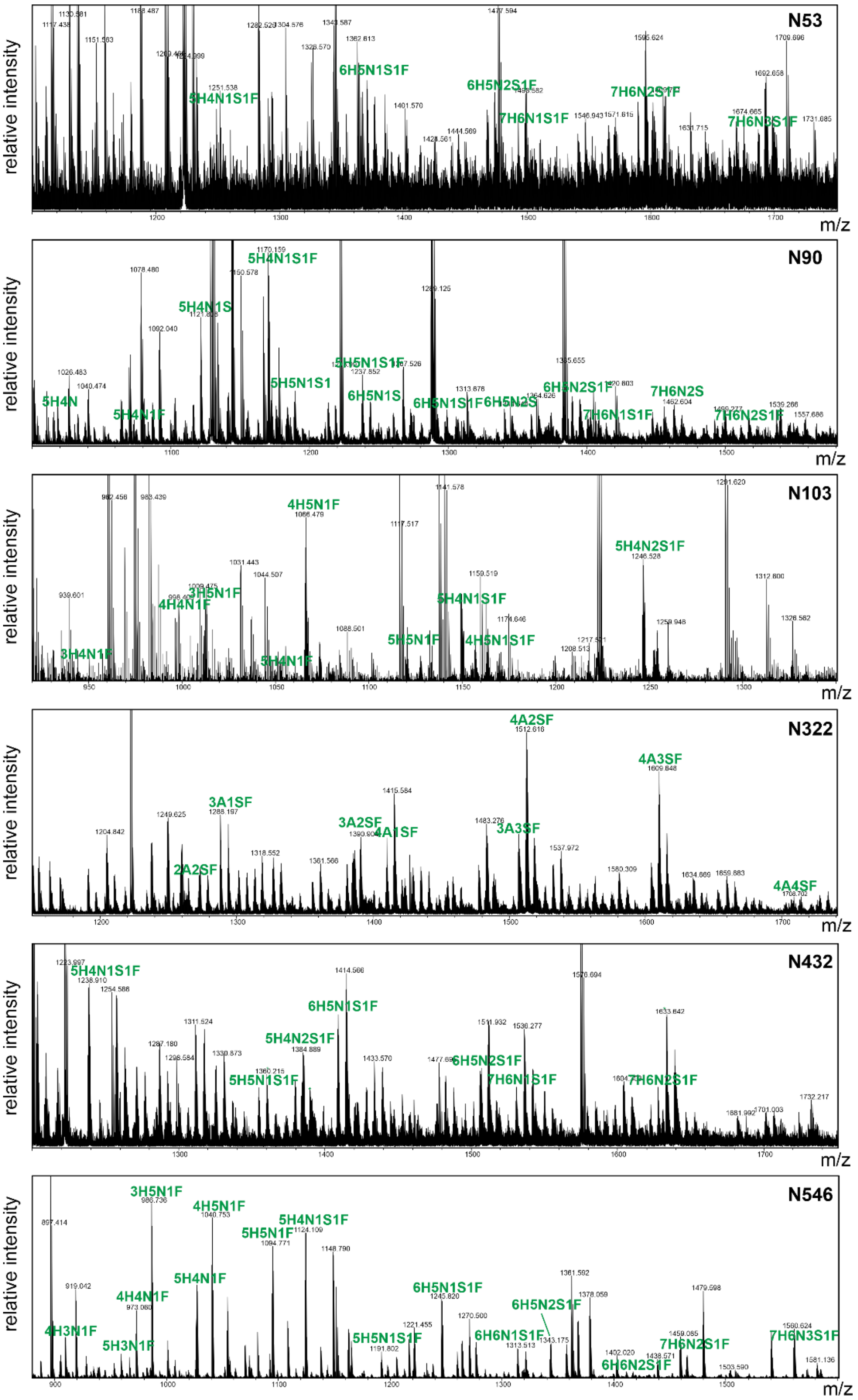

Figure S1. MS spectra $[M+3H]^{3+}$ of glycopeptides from HEK293 produced ACE2-Fc. The peaks corresponding to N-glycan structures were labelled. H, N, S and F represent the number of hexose, N-acetylhexosamine (HexNAc), N-acetylneuraminic acid (Neu5Ac) and fucose residues, respectively.
